# A mixed-methods approach to understanding partnership experiences and outcomes of projects from an integrated knowledge translation funding model in rehabilitation

**DOI:** 10.1186/s12913-019-4061-x

**Published:** 2019-04-16

**Authors:** Jacqueline Roberge-Dao, Brooks Yardley, Anita Menon, Marie-Christine Halle, Julia Maman, Sara Ahmed, Aliki Thomas

**Affiliations:** 1 School of Physical and Occupational Therapy, McGill University, Montreal, QC Canada; 2 Centre for Interdisciplinary Research in Rehabilitation of Greater Montreal (CRIR), Montreal, QC Canada; 3 Centre for Medical Education, Faculty of Medicine, McGill University, Montreal, QC Canada

**Keywords:** Integrated knowledge translation, Partnership, Impact, Rehabilitation, Clinician, Researcher, Funding model, Project outcomes

## Abstract

**Background:**

Integrated knowledge translation (IKT) can optimize the uptake of research evidence into clinical practice by incorporating knowledge users as equal partners in the entire research process. Although several studies have investigated stakeholder involvement in research, the literature on partnerships between researchers and clinicians in rehabilitation and their impact on clinical practice is scarce. This study described the individual research projects, the outcomes of these projects on clinical practice and the partnership experiences of an initiative that funds IKT projects co-led by a rehabilitation clinician and a researcher.

**Methods:**

This was a sequential explanatory mixed methods study where quantitative data (document reviews and surveys) informed the qualitative phase (focus groups with researchers and interviews with clinicians). Descriptive analysis was completed for the quantitative data and thematic analysis was used for the qualitative data.

**Results:**

53 projects were classified within multiple steps of the KTA framework. Descriptive information on the projects and outcomes were obtained through the survey for 37 of the 53 funded projects (70%). Half of the respondents (*n* = 18) were very satisfied or satisfied with their project’s impact. Only two (6%) projects reported having measured sustainability of their projects and four (11%) measured long-term impact. A focus group with six researchers and individual interviews with nine clinicians highlighted the benefits (e.g. acquired collaborative skills, stronger networks between clinicians and academia) and challenges (e.g. measuring KT outcomes, lack of planning for sustainability, barriers related to clinician involvement in research) of participating in this initiative. Considerations when partnering on IKT projects included: the importance of having a supportive organization culture and physical proximity between collaborators, sharing motives for participating, leveraging everyone’s expertise, grounding projects in KT models, discussing feasibility of projects on a restricted timeline, and incorporating the necessary knowledge users. Clinicians discussed the main outputs (scientific contribution, training and development, increased awareness of best practice, step in a larger effort) as project outcomes, but highlighted the complexity of measuring outcomes on clinical practice.

**Conclusion:**

The study provides a portrait of an IKT funding model, sheds light on past IKT projects’ strengths and weaknesses and provides strategies for promoting positive partnership experiences between researchers and rehabilitation clinicians.

**Electronic supplementary material:**

The online version of this article (10.1186/s12913-019-4061-x) contains supplementary material, which is available to authorized users.

## Background

In rehabilitation practice, research evidence is not adequately integrated into clinical practice and consequently, patients may not be receiving optimal care [1–11]. For this reason, many Canadian funding agencies have committed to bridging the research-practice gap by supporting knowledge translation (KT) research projects that focus on moving research into practice, programs and policy. Integrated knowledge translation (IKT), a special form of KT has garnered increasing recognition due to its emphasis on actively involving relevant stakeholders and knowledge users (i.e. patients, clinicians, policy-makers) throughout the research process [12–20]. Existing evidence on IKT indicates that involving stakeholders in a participatory research approach strengthens the evidence that is created and optimizes its uptake in practice [2, 21–23]. More specifically, IKT approaches have been found to increase social workers’ sense of competence, to foster researchers’ and decision makers’ understanding of each other’s roles and to empower stroke patients to participate in community-based exercise [21, 23, 24]. While reviews on participatory research partnerships in public health have shown benefits such as improved quality of outcomes, increased sustainability of project goals over time and the building of professional capacity and competence in stakeholder groups, very few studies have adequately described, assessed and reported on IKT activities specifically involving clinician-researcher partnerships in rehabilitation [3, 17, 25, 26]. There is a need to examine the range of projects, project outcomes and the nature of collaborations between rehabilitation clinicians and in order to advance our understanding of the IKT research process.

The purpose of this study was to describe the process and deliverables of individual research projects within an IKT funding model involving partnerships between university-affiliated researchers and rehabilitation clinicians in Montreal, Canada. The questions that guided this research were:

1. What was the nature and scope of funded projects and the phase of the KT process addressed by each?
2. What were the outcomes of each research project on clinical practice?
3. What were the project leaders’ and principal investigators’ perceptions of the partnership experience?

## Methods

### Context

The School of Physical and Occupational Therapy at a research-intensive university in Quebec, Canada receives funding from the Richard and Edith Strauss Canada Foundation to support a bi-annual call of proposals to fund IKT projects for a one-year period. Since 2009, the Foundation has funded more than 53 IKT research projects within the field of rehabilitation. These projects involve a collaboration between a university-affiliated researcher (principal investigator or PI) and a clinician (project leader or PL) with the goal of collaborating to bridge the research-practice gap and improve patient outcomes and quality of care. To date, the collective effect of these IKT research projects has yet to be reported.

### Outcomes (operationalization)

For the purpose of this paper, the word *outcomes* will be defined as the intended and unintended consequences of an event, process or program [27] from the perspectives of the research participants and should not be confounded with study outcomes or health outcomes (i.e. clinical or economic outcomes).

### Study design

A mixed methods sequential explanatory design was used for this study [28–30]. The two-step quantitative phase consisted of 1) a document review to characterize the nature and breadth of each project and to identify the alignment of each project’s aim with the KTA framework; and 2) a survey on the characteristics and outcomes of the funded projects. The qualitative phase consisted of focus groups with PIs (researchers) and interviews with PLs (clinicians) to explore their perceptions of project outcomes and their experiences with the partnership. Ethics approval for this study was obtained from the McGill University Institutional Review Board.

### Quantitative phase

#### Step 1: document review

For each funded project the research team obtained the following information from the ES steering committee files: project title, project abstracts and PIs’ and PLs’ names and work positions. Two independent coders mapped each project using titles and abstracts onto the Knowledge-to-Action (KTA) framework’s [31, 32], knowledge creation funnel (knowledge inquiry, knowledge synthesis and knowledge tools or products) or action cycle step: 1) identify the problem, 2) adapt knowledge to local context, 3) assess barriers or facilitators to knowledge use, 4) select, tailor, implement interventions, 5) monitor knowledge use, 6) evaluate outcomes, 7) sustain knowledge use. For example, a project that identified the barriers related to implementing an evidence-based treatment was coded as “*Step 3 of the KTA cycle - assess barriers to knowledge use*.” Disagreements were resolved by full-text readings, discussion and input from a third reviewer.

#### Step 2: survey

##### Development.

The research team developed a 20-item survey to gather descriptive information on the nature of the projects, the outcomes and the partnerships (see Additional file 1). The survey consisted of six open-ended items, 12 closed-ended items and two items scored on a 5-point Likert scale within four sections: 1) general information, 2) study outcomes, 3) impact and sustainability, and 4) reflection on the partnership. For instance, two open-ended questions were designed to elicit participants’ perceptions of both the project’s expected impact and actual impact. Seven members (clinicians and researchers who are experts in KT) of the ES steering committee then reviewed it for clarity, length and appropriateness of the items. Revisions were made accordingly. The survey was administered online using LimeSurvey [33].

##### Participants and recruitment.

The research team contacted the respective PIs from projects funded between May 2009 to August 2015 via email to solicit their participation in the online survey. A reminder email was sent two weeks later.

#### Data analysis

Descriptive statistics including frequencies and percentages were used to summarize the data from the close-ended questions (e.g. study setting, target group). Two student researchers (professional master’s students in occupational therapy, supervised by a PhD professor and expert in KT research) independently coded open-ended responses using conceptual content analysis [34]. Discrepancies were resolved by face-to-face discussion and a third reviewer, who independently coded the select open-ended responses, was invited for final consensus.

Congruent with the analytical process of mixed methods sequential designs, the quantitative findings were used to inform the interview and focus group guide [28]. In this study, the linking of quantitative and qualitative data occurred at the design-level, where results from the first phase were used to build the second phase of research design, at the method-level through connecting, building and merging the databases and at the interpretation and reporting level through the continuous approach [35].

### Qualitative phase

#### Methodology

An Interpretive Description (ID) methodology was used for this phase of the study. ID allows researchers to construct interpretations that may be useful in guiding practice in a particular time and context [36, 37]. This focus on the practical and the locally constructed reality was deemed appropriate for the purpose of reporting on the outcomes of this funding model, ultimately with the aim of developing recommendations to increase the impact on practice.

#### Participants and recruitment

The eligible PIs and PLs involved in the 53 projects were invited by email to participate in a focus group or interviews, respectively. Eligible PLs were clinicians who co-led a project that ended in March 2015 or earlier, to ensure that they had sufficient time to observe the impact of their project. Purposive sampling of PLs was used to ensure heterogeneity of perspectives on issues such as satisfaction with impact, satisfaction with the partnership, and measurement of sustainability.

#### Procedure and data collection

##### Focus group with PIs.

A focus group guide was developed based on the results of the quantitative phase (see Additional file 2). A member of the ES steering committee provided feedback on the guide regarding clarity and relevance. Two research assistants with previous experience in facilitating qualitative interviews moderated the 90-min focus groups. Discussions were audio-recorded and transcribed verbatim.

##### Interviews with PLs.

Semi-structured interviews were conducted with PLs because their experiences and backgrounds were more heterogeneous than those of researchers’ and the one-on-one format would allow for discussion of potentially sensitive matters such as the clinical environment. The interview guide (see Additional file 3) was developed based on results of the quantitative phase and the focus group with PIs and contained questions exploring PLs’ perceptions regarding their researcher-clinician partnership and project outcomes. The interview guide was pilot-tested with a member from the ES Steering Committee and changes were made accordingly. Two members of the research team facilitated the interviews which lasted on average one hour, were audio-recorded and transcribed verbatim.

#### Data analysis

Data collection and preliminary analyses were co-occurring. Transcripts were coded independently by two researchers who piloted the coding techniques on an interview to establish a similar coding frame. The analytical process included a constant and iterative review of the data, guided by questions aimed at identifying coherent narratives and themes [36, 38]. An open coding process was done at the paragraph and statement level of the transcripts (see Additional file 4). Open codes were then grouped into overarching themes. Visual representations of the codes and themes were arranged onto storyboards. Numerous iterations of analysis were performed, which involved repeatedly asking questions such as “what is going on here?” and “what am I learning about this?” in order to identify coherent narratives rather than lists of descriptive categories [38]. Analysis was concluded when all members agreed that the coding book represented the data collected.

## Results

### Document review

**Fig. 1:**
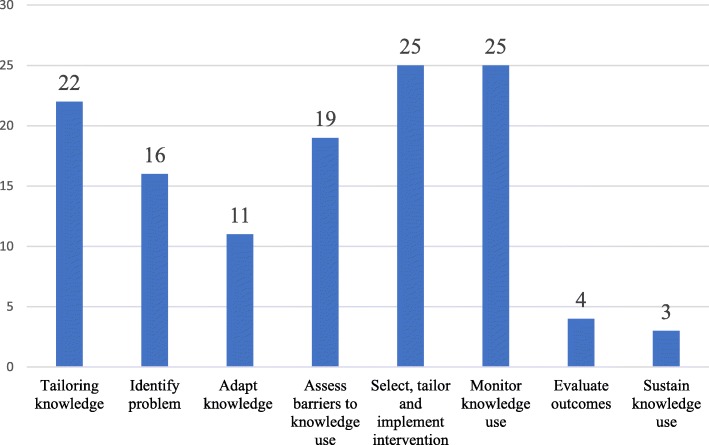
Number of studies classified in each step of the KTA framework; a mapping of the funded IKT projects on the KTA framework

The 53 projects were classified onto the KTA framework (Fig. 1) with 40% of studies targeting two steps of the KTA cycle, 26% onto one step, 17% onto three steps, 11% onto four steps, 4% onto five steps and 2% on seven steps. The most frequent KTA steps were 4 (*select, tailor and implement intervention*) and 5 (*monitor knowledge use*) of the action cycle with 25 studies having been mapped onto each step. Steps 6 (*evaluate outcomes*), with only four studies, and 7 (*sustain knowledge use*) with three studies, were much less commonly examined. Work positions of the 53 PLs were as follows: 53% were clinicians, 26% were Master’s of Science (MSc) candidates, and 21% were Doctoral (Ph.D.) students or Postdoctoral fellows.

### Survey

Thirty-seven of the 53 eligible projects (70%) were accounted for in the survey. Table 1 presents general information on the projects (e.g. design, setting). Twenty-two studies (59%) focused on health care professionals and represented a variety of professions. Twenty-eight projects (76%) used a KT conceptual framework and of those, 24 (80%) used the KTA (Table 1). Of the nine projects that did not use a KT framework, two projects were carried out during the first year of the initiative when this was not a requirement of the call for grants.

**Table 1:** Survey results - general information

*N = 37*		n
Project Status	Completed	29
	In progress	8
Study Design	Qualitative	17
	Quantitative	11
	Other	9
	Mixed Methods	8
Study Setting	Rehabilitation	16
	Other *(community-based* (5)*, university* (3)*, national or international network of clinicians* (3)*, communities of practice and school district)*	13
	Acute care	7
	N/A	5
	Tertiary care	3
Target population	Health care providers	22
	Adults (18–65)	12
	Other professionals *(managers* (2)*, administrative staff* (2)*, educators in rehab ethics, students, faculty, elementary school teachers*)	8
	Older adults (65+)	5
	Parents or caregivers	4
	Other *(researchers, university students, decision makers)*	4
	School-aged children (4–12)	3
	Adolescents (13–17)	2
	Community/NGOs/grassroots	1
	N/A *(scoping review)*	1
	Infant/toddler (0–3)	0
Health condition of study population	No specific condition/mixed	10
	N/A *(health care providers* (4)*, scoping review))*	5
	Chronic pain	4
	General mental health	4
	Stroke	3
	Back pain (including neck)	3
	Cerebral Palsy	2
	Rheumatoid arthritis/Osteoarthritis	2
	Hip fracture	1
	Autism Spectrum Disorder	1
	Spina Bifida	1
	Multiple Sclerosis	1
	Osteogenesis Imperfecta	1
	Burn survivors	1
Stakeholders involved in the project	Health system/care practitioners	28
	Health System/Care Managers	15
	Patients/consumers of health system/care	14
	Families/caregivers	7
	Health System/Care Administration	7
	Students	6
	Health System/Care Professional Organizations	4
	Federal/Provincial Representatives	2
	Community/Municipal Organizations	2
	Consumer Groups/Charitable Organizations	2
	Others (professors, teachers)	2
	Media	1
	Industry/Corporation	0
Use of a KT framework	Yes	28
	No	9
Name of framework or conceptual model used *(N = 28)*	KTA cycle	22
	Theoretical Domains Framework	2
	KTA cycle + Technology Acceptance Model	1
	KTA cycle + Consolidated Framework for Implementation Research	1
	Ottawa Model of Research Use	1
	Transtheoretical model of health behavior change	1

Table 2 summarizes the primary outcomes and associated measurement methods. The most common primary outcomes were clinician practice behaviors, clinician knowledge and clinician attitudes towards evidence use. Individuals (*n* = 4) who selected “not applicable” were conducting a review or developing an instrument.

**Table 2:** Survey results: primary outcomes and measurement methods

*N* = 37		n
What is the primary outcome measured?	Clinician practice behaviors	19
	Clinician knowledge	17
	Clinician attitude towards evidence	14
	Process evaluation	10
	Knowledge dissemination	8
	Stakeholder engagement	7
	Patient outcomes evaluation	4
	N/A	4
	Community attitude towards evidence	4
	Patient knowledge	3
	Other practice change	2
	Other	2
	Patient attitude towards evidence	1
	Informing policy	1
How was the primary outcome measured?	Questionnaire	22
	Focus group	12
	Semi-structured interviews	12
	Other	9
	Standardized Outcome Measure	3
	Concept mapping	0

Four studies (11%) measured long-term impact, and did so at either three months (*n* = 2), nine months (*n* = 1) or one year (n = 1) after project completion. Of the 33 studies that did not measure long-term impact, nine reported that it was “not applicable”. Only two studies (6%) reported measuring sustainability.

Table 3 includes the level of satisfaction with the project’s actual impact, dissemination methods and researchers’ level of satisfaction with the researcher-clinician partnership. Half of the respondents (*n* = 18) were very satisfied or satisfied with their project’s impact. Study findings were commonly disseminated through scholarly conferences (*n* = 24) or publications (*n* = 18). Two-thirds of PIs were very satisfied with the partnership with the clinical site.

**Table 3:** Survey results: Satisfaction with impact, dissemination of results and reflection on the partnership

*N* = 37		n	%
Level of satisfaction with the actual impact of your project	Very satisfied	7	19
	Satisfied	11	30
	Somewhat satisfied	6	16
	Neutral	5	14
	Not satisfied	1	3
	*Did not answer*	*7*	*19*
How were the results of the project disseminated?	Scholarly conference	24	
	Publication	18	
	In-service workshop in your clinical setting	7	
	Further funding	6	
	Graduate course	5	
	In-service/workshop in another clinical setting	4	
	In-service/workshop in your research setting	3	
	Web-based resource	2	
	Material	1	
	Non-peer-reviewed paper	1	
	*Did not answer*	*6*	
Level of agreement with the statement: *I am very satisfied with the partnership I formed with the clinical site*	Strongly agree	16	43
	Agree	9	24
	Somewhat agree	3	8
	Neutral	7	19
	Somewhat disagree	0	0
	Disagree	1	3
	Strongly Disagree	0	0
	*Did not answer*	*1*	*3*

**Fig. 2:**
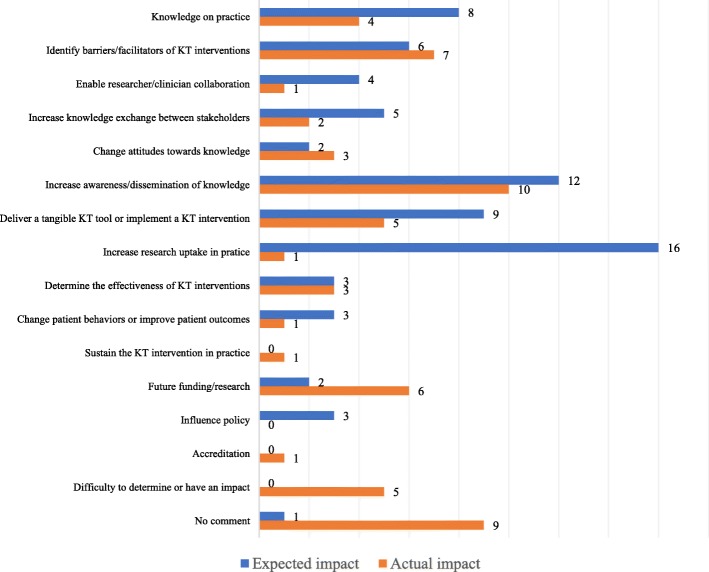
Principal investigators’ expected vs actual project impact; a comparative bar graph displaying what principal investigators expected to see as an impact of their research project versus their perception of the project’s actual impact

Figure 2 represents a comparison of respondents’ perceptions regarding expected versus actual project impact. For eight of the 16 reported outcomes, the actual impact fell short of the expected impact.

### Focus group with principal investigators

Nineteen eligible PIs were invited for a focus group. The focus group consisted of six rehabilitation researchers who were full (*n* = 1), associate (*n* = 1) or assistant professors (*n* = 4) at the University where the research was conducted. Results are organized under four overarching themes: 1) individual projects are framed within a broad vision of research; 2) we can’t measure everything; 3) ambiguity around defining success; and 4) building partnerships with clinicians and clinical program directors (see Table 4 for themes, nested categories and corresponding quotes).

**Table 4:** Results of the Focus Group with Principal Investigators

Overarching themes	Nested categories	Excerpts
Individual projects are framed within a broad vision of research	A project is a piece of a larger research project	Research is on the 5 to 10-year plan, so [projects] are just pieces of bigger programs for which the funding was extremely useful Well they are taking a piece of a larger project With one year projects, if you don’t continue on, there is no way to have any long-term or very extensive evaluation of impacts
	KT framework as essential	It needs to be framed within a larger project where this creation of knowledge will serve to be implemented You need to word it in a knowledge translation framework […] You need to frame it in terms of knowledge translation
	Planning ahead for sustainability	I would take that end part of the grant application where you look at future directions very seriously, as opposed to saying a couple publications here. Consider whether a publication is really the end point of this. If you don’t know where you’re going with your project, you’re not going to get there. So, you have to design the project from the end and sort of say where do I want to go and what piece is this. We already know that we have to think about sustainability right from the beginning or at least where its going to lead in the future. It basically died after the funding period ended […] So, sustainability is a real issue.
We can’t measure everything	Lessons learned by researchers	This is research, not everything is going to work out, but if you don’t try, and the lessons learned, with all due respect, you learn those lessons even if you apply those in 5 years […] You learned how to engage a group, the continuity is in you, that is where the continuity is. There are learning lessons also that come from, at least from one of the projects where it was very challenging to recruit. They help plan future projects that we want to take. It is part of a larger project, so these are actually very useful for us to learn and improve on what we are doing
	Project leader as an agent of change	What is not really measured is the change in attitude and their engagement. I think it is very significant and I’m quite sure would have significant and important impact in their respective practice with their colleagues [Clinicians] feel more empowered to changing.
	The unknown impact of dissemination strategies	There are a lot of outcomes that may not be so tangible that we may not continue to measure and we don’t know whether things have changed, attitudes or practices that have been implemented in different ways. A lot of what I’ve learned in these projects has informed students in my teaching.
Ambiguity around defining success	Success	That may be actually an important aspect in terms of judging the success of the project, as where the intended impact is expected to happen. I mentioned I was involved in a current project the goal was to produce a meta-analysis. There I would say, publication of that data would be a successful project because the goal is not to impact a specific group of clinicians at a specific centre, but to distribute a certain better analyzed knowledge wide It’s not necessarily a project that gets finished but a project that leads to something more. So I think it was successful in the sense it was published and we understand, we moved forward. But not yet successful in the way that we really changed practice. It was just the beginning. So successful, ‘yeah, we published and understood’. But not yet, ‘yeah I’m happy I’m done, we are just starting’. If a project has an ending, I’m not sure it’s a successful knowledge translation project.
	Failures	I thought it wasn’t successful because the student did not finish and did not produce publications The project I was involved in was not necessarily successfully even if we reached all the goals we had set out, simply because there was no follow-up to those goals. There is no such thing as failure. Because...The whole [research program] is not going to fall apart because of your one study
Building partnerships with clinicians and clinical program directors	Clinical context as a significant driver	Right now, the current funding, the way things are, you don’t have time for research. […] you just see so many patients a day and that’s the reality. [clinicians] can have ideas, but you know they are not going to have the time free to do it. The system just doesn’t allow it. These higher level structural organizational kinds of things do make a big difference and can really help get clinicians on board. We used to have a salary award by foundation, this has been suspended for two years now. But the salary award program was very successful.
	Understanding clinician motivation	Engaging with clinicians, engaging with other partners, I think that as a group, we need to think about what’s in it for them. Like what are the people going to get out of it that’s going to make it sustainable, and you know, participation, and you know, that sort of thing, and making it clear on that. This is called motivation, it is the effort against the outcome. So maybe the outcome was not high enough for the effort. I think we have to kind of think of that. In terms of how much effort are we asking our people to do and what is the outcome for them.
	Feasibility of projects co-led by clinicians	The questions that are being raised by clinicians are pretty unviable. And you know the methodology that you need to ask them is like impossible, like beyond the scope. They tend to be asking impossible questions, like questions that are very high level […] almost policy level questions. That’s part of the disconnect between what they want to get out of it and the reality of what they can achieve.

#### Theme 1: Individual projects are framed within a broad vision of research

This theme describes how each project was seen as being part of a more expansive program of research.

##### A project is a piece of a larger project

Researchers spoke about leveraging the Strauss funding to advance programs of research that went beyond the one-year timeline, which only allowed for a portion of a research program to be developed.

##### KT framework as essential

Researchers reported that regardless of a project’s place on the KTA cycle, projects must be informed by a KT model in order to optimize the success of future implementation efforts.

##### Planning ahead for sustainability

Researchers explained the need to set clear objectives and strategies for sustainability of the innovation. The group agreed that sustainability was not sufficiently considered during the planning stage of these projects but was necessary to reach the ultimate end goal.

#### Theme 2: We can’t measure everything

Some outcomes of the research projects are difficult to measure as they impact people in varied and complex ways that are not explicit and quantifiable.

##### Lessons learned by researchers

Researchers gained valuable lessons from participating in these projects such as strategies to better recruit participants or work with clinicians; these lessons could be quite impactful in advancing subsequent projects. These lessons, however, are not easily quantifiable as the information gained could be stored and used in different ways for different people.

##### Project leader as an agent of change

Researchers expressed that the clinician PLs with whom they had partnered were more likely to use and apply research in their own practice, were more inclined to apply for future research initiatives, felt more empowered to influence colleagues in applying best practice and were more comfortable in their roles as evidence-based clinicians.

##### The unknown impact of dissemination strategies

Researchers reflected on the effect of their project’s dissemination strategies and believed that presentations, publications or posters have an important impact on end users. However, they added that the extent to which these dissemination methods actually affected the target audiences on the short and long-term was challenging to measure explicitly.

#### Theme 3: Ambiguity around defining success

Researchers agreed that the success of the projects was relative rather than absolute; success was dependent on whether the knowledge acquired from the project contributed to advancing the research program. In light of this account, even those projects that were generally considered failures (i.e. those that were abandoned or had no follow-up), could be successful as they provided knowledge informing subsequent projects and contributed directly or indirectly to the continuity of the research program. Researchers reported a distinction between the success of one single project, considered successful if it achieved its specific research objectives or if it led to something more and advanced the research program vs. the success of entire research program. The latter was considered successful if it was sustained.

#### Theme 4: Building partnerships with clinicians and clinical program directors

Researchers described certain considerations they believed were important to understand and respond to when partnering with clinicians.

##### Clinical context as a significant driver

Organizational structures have a major role in shaping the IKT research process acting as either a facilitator or barrier. The main challenge in creating meaningful partnerships with clinicians was their lack of dedicated time for research. A supportive organizational structure (e.g. salary award program) was believed to optimize clinician engagement in research activities.

##### Understanding clinician motivation

Clinicians’ motives for participating in research was important to consider as varying sources of motivation (i.e. intrinsic or extrinsic) could impact the outcomes and viability of projects. For example, clinicians who proposed a research question to address a clinical problem were more likely to be actively involved in the IKT research process and this could, in turn, ensure the project’s sustainability.

##### Feasibility of projects co-led by clinicians

Researchers described a disconnect between what clinicians aimed to get out of a research project and the reality of what could have been achieved in a research context. This resulted in clinicians posing research questions that were not always feasible.

### Interviews with project leaders

Fifty-one PLs were invited for individual interviews. Nine individual interviews (18%) were conducted with six rehabilitation clinicians and three graduate students (MSc or Ph.D.). Data from one Ph.D. student PL were excluded from the analysis as the participant self-identified primarily as a researcher and could not answer questions regarding the impact on clinical practice. Participants had an average of 20 years of clinical experience (ranging = 8 to 36). Two of the eight PL participants did not have research experience before participating in their research project.

Analysis of interview data revealed three overarching themes: 1) project deliverables; 2) exploration of partnership dynamics; and 3) facilitators of effective IKT (see Table 5 for themes, nested categories and corresponding quotes).

**Table 5:** Results of the Interviews with Project Leaders

Overarching themes	Nested categories	Excerpts
Project Deliverables	Scientific contribution	Two articles, I did 4–5 oral presentations and an international conference, a poster presentation. There was something produced now that is available on YouTube and Internet site that physios, OTs and the general population can see.
	Training and development	It demystified what research was and made it more accessible. I feel more confident answering patients’ questions. I feel more confident in my abilities to go get the information. Being an agent of change in terms of pushing the team here, we all know there’s a positive to doing it, but now I have the back-up, the information to back it up. It’s also the people that you meet and you’re growing your network. So, I think that helped my career. I don’t know if it’s a career, but it did help my academic CV to improve.
	Increased awareness of best practices	Now there’s more formal kind of processes in place to incorporate the knowledge and the expertise of the patients into decision-making. It had an impact on the psychologists and physicians I work with, […] the overall care of the team, I like to say, has changed. We didn’t change the world, but maybe it started to bring ideas or reminded physios that this was a hot topic and something to think about. Awareness increase, that could be my outcome. The awareness of clinicians was a bit greater after the project. Clinical outcomes are hard… for the clinical side, I have to say, it didn’t change much.
	Step in a larger effort	Getting the pilot, like a small part of the project started, so after you can have some data to show to bigger grants, so, a first step, a good first step. I feel like I just did one piece on a big puzzle that is way too big to handle by myself.
	Difficulty measuring clinical changes	It’s hard to say sometimes, because you never know, people what they’re gonna take with them and how they’re going to apply that in their clinical practice. I brought the results of our scoping review and shared our results with the participants. Did they integrate that knowledge? Did it lead to a change in their practice? I don’t know for sure. Change does not happen within three months, only within a year, two years, and we don’t have any way to capture that. You don’t have time to grow with the project.
Exploration of partnership dynamics	Shared leadership	I think it was a good partnership because I brought my reflection, my mind, my reflexivity from clinical practice. And they were willing to help me out with the research background, to make sure my research was well-thought and would be strong research proposal. Power issues were related to hierarchies that emerged based on perceived education, knowledge, status of different stakeholder.
	Researcher as the leader	So when you don’t know the process, you need somebody to guide you and that’s what the mentor was there for, for me. Guidance. Every step of the way. Everything was discussed with me, but I didn’t coordinate what was going on […] I didn’t have to worry about… all the questions about feasibility and reliability. Everything was dealt with by the research department, with the researcher.
	Clinician as the leader	I was technically the leader, so as soon as she suggested the idea, I was definitely on board and I was always referring to her as a consultant. It was kind of a tacit agreement that I was responsible for the project and I was to approach them with questions.
	Ideal partnership	If you can use their expertise, that’s going to motivate them. […] And just knowing that the researchers will be there to support them in what is not their area of expertise. A straight goal from the start, like everyone knew what we were doing. […] what are the goals? What are the resources? What are barriers? What are the actual facilitators? And the timeline of the project. So everyone knew exactly, where we were going, and I tried to detail their contribution individually, from the start, to make sure that everyone knew their role in the project. An ideal partnership is one where […] people are excited about doing it. It’s meaningful to them.
Facilitators of effective IKT	Being motivated to participate in research	[The PI] was the head of that committee at the time. So I just go to her and say, well this interests me if you have anything that comes up in the future. I wanted to offer the best services possible, I wanted to develop something that would be useful to somebody. “What makes me all excited is the tangible outcome that is at the end [...] to take that and then to apply that into clinical practice.” I was also interested in the research part and that was my incentive to be so invested in the project. If you’re not really interested in the research world, […] I can see why it would be challenging.
	Institutional support	I think the motivation of clinicians is there, but the structure of the environment, the working environment doesn’t allow them to get involved in research. So, if you have, if the institution allows for research and allows time and facilitates this for clinicians, then it’s a win-win situation.
	The proximity of researchers	To have researchers on the premises, [...] around you, around your environment, for me was a big positive, was a motivation. Having a researcher right next to your office is gold […] closeness is a key.
	Previous research experience	I think clinicians need to get used to research for it to be interesting, and not to be too much intimidating. [previous research experience] gave me the motivation, the bug for research. After that, it was like, oh okay let’s do more research

#### Theme 1: Project deliverables

Project leaders discussed the main outputs as a result of the work done during their projects.

##### Scientific contribution

PLs spoke about the project’s impact in terms of knowledge generated for the scientific community and its dissemination through scientific publications, presentations and posters at conferences, universities, and clinical sites and through YouTube videos and websites.

##### Training and development

PLs reported that they improved their research-related skills and felt more competent as collaborators on an IKT research project, as evidence-based practitioners and as change agents in their clinical settings. Participation in the initiative developed PLs’ social networks, enhanced existing partnerships with researchers and strengthened clinicians’ academic resume which would potentially help them gain further funding and research opportunities.

##### Increased awareness of best practices

Most PLs reported an increased awareness of evidence-based practice (EBP) in their clinical site as a result of their involvement in the IKT project.

##### Step in a larger effort

PLs discussed how a project was a stepping stone to a larger research program which often enabled them to be more competitive for external funding. PLs believed the impact of their projects should be considered on a larger scale.

##### Difficulty measuring clinical changes

Though clinicians admitted that their own practice had changed considerably since participating in the projects, statements on actual clinical changes were somewhat vague. PLs reported that measuring the impact on patient and practice outcomes was complex because of the variety of dissemination methods and stakeholders involved. They believed that the overall impact was positive but could not quantify it. PLs also commented on the insufficient amount of time (i.e. one year) for measuring clinical changes, impact and sustainability.

#### Theme 2: Exploration of partnership dynamics

Clinicians discussed the three main types of partnership dynamics they encountered and their views on an ideal partnership.

##### Shared leadership

Four of the PLs interviewed revealed that decision-making was equally shared between themselves and the researcher. Three PLs expressed no difficulties with power sharing and alluded to an implicit role assignment aligned with each person’s expertise. The other PL reported challenges with shared leadership; they expressed feeling confused about roles and struggled with power sharing between themselves and the PI.

##### Researcher as the leader

In these two projects, the PLs had no prior research experience and needed support in understanding research and their role within the research enterprise. One PL was always part of the process and referred to the researcher as a “mentor”; the other admitted to being less engaged in the overall research process and to not having opportunities to influence its course.

##### Clinician as the leader

The two PLs who reported taking the actual lead on the project were the two graduate students. They were responsible for all details of the research process and for ensuring sustainability of the project.

##### Ideal partnership

An ideal partnership was one where everyone’s expertise was leveraged, where expectations, goals and roles were clear and where the work was meaningful for all those involved.

#### Theme 3: Facilitators of effective IKT

Project leaders commented on the conditions which impacted their engagement and participation in the research project.

##### Being motivated to participate in research

Clinicians revealed that motivation to participate in research was crucial in ensuring a positive IKT experience. They listed factors such as vested interest in a specific research topic, desire to improve clinical practice, and personal interest in learning and doing research as reasons for participating.

##### Institutional support

Clinicians explained that having a supportive organizational structure that allows for time, funding and resources for research would optimize their interest in research, participation and engagement. They reported that the current working environment was discouraging and impeding their involvement in research.

##### The proximity of researchers

Physical proximity to researchers was a facilitator for clinician engagement in research. Clinicians suggested that frequent and casual in-person meetings optimized the partnership.

##### Previous research experience

Clinicians with research experience or those with graduate level education felt more comfortable participating in a research project than those without research experience.

## Discussion

This study sought to describe the outcomes of IKT research projects involving a rehabilitation clinician and a researcher. In doing so, it provides a portrait of IKT projects, their outcomes, the facilitators and barriers to creating partnerships between researchers and clinicians and the challenges in measuring and sustaining IKT projects.

### Evaluating outcomes

The variation in types of projects and targeted steps of the KTA cycle brought about a wide range of outcomes. The survey showed that the most popular primary outcomes measured were clinician practice behaviors, clinician knowledge and clinician attitudes towards evidence whereas patient-oriented outcomes were much less popular with only 8% of studies examining patient knowledge and 3% of studies examining patient attitudes towards evidence. The qualitative phase revealed an unintentional yet important effect on clinicians’ sense of competency as change agents in their settings and as evidence-based practitioners. This aligns with previous IKT work emphasizing the professional capacity and competency building developed in stakeholder groups [25, 26, 39].

Our findings indicate that actual impact fell short of the expected impact, which is incongruent with PIs’ and PLs’ reporting of moderate/high satisfaction with impact. Researchers and clinicians were generally unable to provide concrete sustained clinical or health care system outcomes as a result of their project but were confident that steps towards best practices were made. Tangible outcomes of a single project will vary, but most projects are likely to advance the researcher’s agenda forward and/or increase clinicians’ awareness and attitudes towards EBP. Though some projects may fail to meet their objectives, the knowledge and experience gained from the project may inform follow-up projects. While this study provides an initial portrait of the projects from this IKT funding model, it emphasizes the need to develop a robust measurement framework with a comprehensive set of indicators (e.g. types of knowledge use, health, provider, organizational) early in the planning phases, psychometrically sound measurement tools and appropriate measurement time points [31, 40].

### Impact and sustainability

Graham and colleagues operationalize impact into *health/patient, practitioner/provider* and *system/societal* outcomes [31, 40]. Our survey findings demonstrate that a modest number of projects (11%, *n* = 4) reported measuring long-term impact with one project changing *health* outcomes and no projects reporting on *provider* or *system* outcomes. However, participation in these projects influenced clinicians’ personal use and application of knowledge (*provider* outcomes) such as attitudes towards EBP and EBP behaviors, which in turn, influenced their colleagues’ knowledge use and application. Challenges in measuring impact were noted and included short time-frame for the projects, being smaller pieces of a broader project, and difficulty measuring outcomes. Overall, clinicians and researchers reported that their projects still had an impact on the uptake of research in practice, whether it was direct or, more often, indirect, as it propelled the research agenda forward.

*Sustainability*, defined as “the degree to which an innovation continues to be used after initial efforts to secure adoption is completed”, is the last step in the KTA cycle and it is often assessed two or more years after initial implementation [40, 41]. This could explain why only two projects reported measuring it. The allotted one-year timeline and funding envelopes supporting these projects may limit sustainability activities, a finding also revealed in a scoping review on partnership engagement in rehabilitation research [3]. Our results suggest a critical need for project collaborators to adequately plan for sustainability regardless of the project’s alignment with one of the seven phases in the KTA cycle. This aligns with recommendations for researchers and collaborating stakeholders to consider the boundaries and scalability of their KT intervention [40, 42]. IKT project proposals could include sustainability-focused models and should consider the following factors: relevance of the topic, benefits, attitudes, networks, leadership, policy articulation and integration, financial and political [43–45]. Sustainability is a complex concept that involves multiple levels of consideration such as, its conceptualization, the desired impact and stakeholder goals, choosing an appropriate timeframe, examining fidelity and adaptation to changes, and measurement [42]. Sustainability is a critical component for the successful uptake of research into practice and as such, requires robust and strategic preparation.

### Bridging of two worlds: research and clinical practice

This study suggests that the IKT initiative has begun to make an important contribution towards bridging the research-practice gap, mainly by training both clinicians and researchers in IKT and helping to shift the research culture towards one that embraces research-practice partnerships early in the research process. Both groups reported having gained valuable skills and knowledge to support future collaborations, which aligns with the major tenets of the principles of the scholarship of practice [2, 46, 47]. This is an important finding, as previous work has reported lack of skill preparation as a barrier to engaged scholarship [15].

The relationships developed between researchers and clinicians as a result of this initiative may, in fact, be an important outcome and precursor to an ongoing and future change in clinical practice. These research-practice networks form early-stage partnerships which contribute to building trust and the creation of sustainable partnerships [26] and can be considered instrumental resources for successful knowledge sharing [48]. Kothari and Wathen discuss the implications of collaboration in terms of group-level identity transformation and suggest that something “more” is created in addition to tangible end-products as a result of IKT collaborations [25].

Our study has identified a number of factors that facilitate researcher-clinician collaborations namely nurturing the relationship early in the research process, understanding everyone’s motivation for participating, initiation of a research question by the clinician, aligning roles with expertise, establishing clear expectations from the start, supporting trust and mutual respect, having prior experience with IKT collaborations and being in close proximity to clinicians or researchers. These findings align with numerous studies that examine IKT collaborations [2, 3, 12, 31, 39, 49–53].

### Existing tensions within a researcher-clinician partnership

Our findings reveal a tension between the priorities of researchers to build sustainable and robust programs of research and the realities of working clinicians who face organizational barriers that appear to interfere with the sustainability of research projects. In response to the reported organizational barriers such as lack of allotted time and funding for research, clinicians and researchers in our study suggested facilitators such as protected time for research activities, financial incentives, and an emphasis on quality versus quantity of patient care within the organization. These results resonate with previous work on the organizational structures required for the uptake of research in clinical practice [54] and on instilling a culture of research in clinical environments [55]. Moreover, while clinicians and researchers share the goal of integrating evidence into practice to improve health outcomes, our findings suggest a disconnect between the project ideas proposed by clinicians and the reality of what can be achieved in a research context. However, as per a “true” IKT model, practitioner project ideas reflect clinical reality. Our results bring to light an important, yet missing piece to the majority of these IKT partnerships. Decision-makers, when appropriate, should be included in the research process as they are key stakeholders and gatekeepers in assuring the sustainability of the projects. Our results show that only 17% of health care managers and 7% of administrative stakeholders were involved in the projects when 59% of projects targeted rehabilitation clinicians as their population of interest. Identifying the appropriate stakeholders to include in participatory research projects has been reported as a major challenge [56]. As Bowen and Graham advise, it is critical to reflect on the targeted level of decision making, whether it is clinical, program or health/social policy and to determine the phase of decision-making [2]. Greater emphasis on organizational factors and associated stakeholders is necessary to increase research project uptake and viability.

Finally, the qualitative findings suggest that participating clinicians were not all involved from project conceptualization and throughout the research process, which goes against the major principles of IKT. Efforts must be made to ensure proper co-creation of knowledge as per the IKT approach whereby research collaborators are actively engaged throughout the entire research process from development of the research question to ensuring sustainability of the innovation. This also calls into question the participation of MSc or Ph.D. students as PLs in these IKT projects. This implication should be considered since only 53% of clinician collaborators were actually full-time clinicians. Programs that support these IKT collaborations must establish the characteristics of a ‘clinician’ collaborator.

## Implications

Researchers and collaborators must reflect at the beginning phases of a research cycle on the ultimate end goal of their research project and on the knowledge users and gatekeepers who should participate in the full process. A context-specific sustainability plan for the innovation should be explicitly documented and reviewed by the funding agency. We suggest that this plan be grounded in sustainability theories and models, and include a statement on available local resources, relevant policies and the commitment of stakeholders. It is critical for researchers to set specific indicators of success based on intended impact aligned with an appropriate measurement timeframe which will allow projects outcomes to be measured more explicitly.

Funding agencies may want to consider adapting their structure and timelines to allow for partnership development as opposed to project development. A one-year funding envelope cannot ensure thorough completion of the entire IKT research process from developing trusting partnerships to measuring project impact. Instead, the funding opportunity examined in this study allowed for IKT partnerships to thrive within the one-year period and trusted that project collaborators had planned for the continuation of the research program after the secured funding time. This highlights the utmost importance of discussing strategies for project sustainability at the very beginning of the collaboration and ensuring that an action plan is put into place near the project’s end. We suggest that initiatives supporting researcher-clinician partnerships address the organizational barriers related to involving a working clinician. A collaborative approach that leads to mutually beneficial considerations needs to be established. This can include the number of allotted clinician hours, roles of clinical directors or decision makers in the project and solutions for sustainability of the project and the partnership. Funding agencies should consider revising their eligibility criteria to include the participation of clinical directors or decision-makers.

## Study limitations

Given the exploratory nature of this study, it was not possible to identify relationships between outcomes and characteristics of the project or partnership. While coding of the KTA phases was done principally by reviewing applications with thorough project descriptions, some projects were coded using brief abstracts only. The coding is believed to be robust as coders were thoroughly trained in KT and abstracts presented all relevant study information. The survey item about satisfaction with partnership was phrased as satisfaction with the “clinical site,” which, in retrospect, is unclear. It could have referred to the participating institution receiving a KT intervention, for instance, rather than the intended meaning regarding the clinician co-leader. Similarly, the terminology regarding impact (*rate your level of satisfaction with the actual impact of your project*), and sustainability (*was the sustainability of the intervention measured*) was not adequately defined and participants may have interpreted these terms in various ways given the heterogeneity of projects. These terms may have different implications for different project types, such as scoping reviews or tool development studies in comparison to clinical implementation projects. In the future, it would be pertinent to distinguish various aspects of impact for different types of projects. The results regarding expected and actual impact in Fig. 2 represent the impact from the perception of the survey respondents and may not reflect actual impact of the project. Furthermore, three survey items included the “N/A” response choice which diminishes the overall effectiveness of the scale [57]. The responses to these questions should be interpreted with this consideration in mind.

Despite the level of experience of most researchers in the focus groups, one group of six researchers may not have been sufficient to reach saturation. Though participation of graduate students and post-doctoral fellows in IKT projects is substantial and represented 47% of project leaders, their specific contribution compared to that of clinician project leaders was not explored in this study.

## Conclusion

Our findings suggest that these IKT research projects between clinicians and researchers set the stage for valuable research-practice partnerships and build trust and competency for future IKT collaborations. While project outcomes were vague and often did not reach the desired impact, these projects contributed to the advancement of a research agenda. Few studies measured sustainability and many studies encountered challenges in measuring the actual impact of projects due to the methodological complexity of KT outcomes and the one-year project timeline. Participants also reported on the organizational context as a major challenge to the IKT process and advised future collaborations to negotiate allotted time, funding and resources for working clinicians to optimally participate in research activities. There is no doubt that IKT projects have the potential to advance both research and clinical realms, however, the importance of incorporating the necessary knowledge users and developing a robust measurement framework and plan for sustainability of the research program is unmistakable and required to produce concrete and meaningful impact.

## Additional files

Additional file 1: Survey Questionnaire. this document presents the survey questionnaire used for step two of the quantitative phase (DOCX 15 kb)

Additional file 2: Focus group protocol, this document presents the protocol used to facilitate the focus group with principal investigators (DOCX 16 kb)

Additional file 3: Interview protocol, this document presents the protocol used to facilitate the individual interviews with project leaders (DOCX 15 kb)

Additional file 4: Example of interpretive description coding and analysis, this document presents an example of the early coding process and the iterative reflections during analysis (DOCX 16 kb)

## Abbreviations

EBP: Evidence-based practice
IKT: Integrated knowledge translation
KT: Knowledge translation
KTA: Knowledge-to-Action
MSc: Master’s of Science
Ph.D.: Doctoral
PI: Principal Investigator
PL: Project Leader
