# Supplementary material for: A mixed-methods approach to understanding partnership experiences and outcomes of projects from an integrated knowledge translation funding model in rehabilitation
Source: BMC Health Serv Res. 2019 Apr 16;19:230. doi: 10.1186/s12913-019-4061-x (PMC6469130; doi:10.1186/s12913-019-4061-x)
Supplement: Supplementary file 1 — Survey Questionnaire. this document presents the survey questionnaire used for step two of the quantitative phase (DOCX 15 kb) [file 12913_2019_4061_MOESM1_ESM.docx]

Additional File 1: Survey questionnaire

**General information**

1. What was the project title?
2. What was the purpose of the study?
3. What is the project status? □ in progress □ completed
4. What was the study design? □ qualitative □ quantitative □ mixed-methods □ other
5. Identify the setting in which the project was conducted. □ Acute care □ Rehabilitation □ Tertiary Care

□ Other □ N/A

1. What was the target population? □ Infant/toddler (0-3) □ school-aged children (4-12) □ adolescents (13-17) □ adults (18-65) □ older adults (65+) □ parents or caregivers □ Community/NGOs/grassroots □ Health care providers □ other professionals □ other □ N/A
2. What was the health condition of the target population?
3. Did you use a theoretical framework/conceptual model? □ Yes, specify □ No
4. Please select all stakeholders involved in the project. □ Health system/care practitioners/public health practitioners □ Patients/consumers of health system/care □ Families/caregivers □ Health System/Care Managers □ Health System/Care Professional Organizations □ Health System/Care Administration Federal/Provincial Representatives □ Community/Municipal Organizations

□ Consumer Groups/Charitable Organizations □ Industry/Corporation □ The media □ Students

□ Other

**Study outcomes**

1. What is/was the primary outcome that was measured? Select all that apply. □ Clinician knowledge

□ Patient knowledge □ Clinician attitude towards evidence □ Patient attitude towards evidence

□ Clinician practice behaviors □ Process evaluation □ Patient outcomes evaluation □ Stakeholder engagement □ Knowledge dissemination □ Informing policy □ Community attitude towards evidence □ Other practice change, specify □ N/A

1. How was the primary outcome measured? □ Focus groups □ Semi-structured interviews

□ Questionnaire □ Concept mapping □ Standardized Outcome Measure, specify □ other, specify

1. Specify any secondary outcomes and the measurement tool used.

**Impact and sustainability**

1. Was the sustainability of the intervention measured? □ Yes, specify how. □ No
2. State what you hoped this project would achieve.
3. To the best of your knowledge, please describe the actual impact that this project has had.
4. Was the long-term impact of the intervention measured? □ Yes, specify how and the time frame for measuring the impact. □ No
5. Please rate your level of satisfaction with the actual impact of your project. □ Very satisfied

□ Satisfied □ Neutral □ Somewhat satisfied □ Not satisfied

1. How were the results of the project disseminated? Select all that apply. □ Publication, provide citation. □ Non peer-reviewed paper/publication, provide citation. □ Scholarly conference, provide citation. □ In-service/workshop in your research setting, provide name of setting. □ In-service/workshop in your clinical setting, provide name of setting. □ In-service/workshop in another clinical setting, provide name of setting. □ Web-based resource, provide URL. □ Material, specify medium. □ Further funding, specify. □ Graduate course, specify name of institution and course.

**Reflection on your partnership**

1. Please indicate your level of agreement with the following statement: I am very satisfied with the partnership I formed with the clinical site. □ Strongly Agree □ Somewhat Agree □ Agree □ Neutral □ Disagree □ Somewhat disagree □ Strongly Disagree
2. If you have additional comments regarding your experience, please provide these here.
