# Supplementary material for: A mixed-methods approach to understanding partnership experiences and outcomes of projects from an integrated knowledge translation funding model in rehabilitation
Source: BMC Health Serv Res. 2019 Apr 16;19:230. doi: 10.1186/s12913-019-4061-x (PMC6469130; doi:10.1186/s12913-019-4061-x)
Supplement: Supplementary file 2 — Focus group protocol, this document presents the protocol used to facilitate the focus group with principal investigators (DOCX 16 kb) [file 12913_2019_4061_MOESM2_ESM.docx]

Additional File 2: Focus group protocol

**FOCUS GROUP GUIDELINE**

**INTRODUCTION**

**Background of the project**: “You are all here today because you had experiences with the [IKT] initiative. As you know, [the IKT initiative] promotes collaboration between researcher and clinicians. However, the impact of these partnerships has not yet been explored. Ultimately, we wish to explore the perceptions of researchers on the impact of their research projects and on their experience of the partnership.”

**Procedure**: “We would like to reiterate and reassure you that all comments and opinions provided today will remain confidential. Everything said here today will be transcribed, anonymously, and will be used solely for the purpose of coding qualitative data in regard to the project.”

**Introductions:** Introduce yourself and role

- - - Moderator: Guide the discussion, make sure we stay in line with objectives, ensure that every participant has chance to speak,
    - Co-moderator: Take note of main subjects and transcribe on board, summarize discussion points, assist moderator
    - Note-taker: Observe, take notes on topics discussed

**Tour de table**: Name and number of projects in the IKT initiative

**QUESTIONS**

1. **Definition of successful partnership**
   1. How do you define a successful partnership between researchers and clinicians in a project?
   2. What does a successful partnership mean to you in the context of IKT projects?
2. **Experience of partnership** (What does a partnership look like based on their experience (e.g., in term of communication, power, responsibilities)
   1. How has your experience been with partnering with clinicians for your project or projects?
   2. How does your experience compare or not with this idea of successful/ideal partnership?
   3. What were your partnerships like?
   4. What were the challenges and strengths associated with the partnership?
3. **The impact of the partnership** (The link between the characteristics of the partnership and its impact. Topics 2 and 3 will be addressed together because we will want participants to talk about their partnership and their impact. Do we make the distinction between the impact of the partnership on the expected outcomes and other outcomes (that they did not necessarily measured but that they nonetheless observed more informally?)
   1. What were the impacts of your partnership on the outcome of the project?
   2. Did your partnership have other impacts besides on the specific outcomes of the project?  (*can be used as a probe if they’re being superficial).*
   3. Which aspects of your partnership would you attribute to the outcome of the project?
4. **How do successful partnership take place** (for example, if someone describe that their partnership was successful because they had a good communication and that such a good communication positively influenced the outcomes of the project, we would like to understand why they had such a good communication. Was a good communication natural to them? Or they intentionally put forwards strategies to foster a good communication? Or it’s a strength they have in general as researchers? Or they worked with people who were available?)
5. **The evolution of the partnership**
   1. How did the partnership evolve throughout the project, from the beginning to the end?
   2. Are the partnerships sustainable?
6. **Concluding questions/summary/recommendations**
   1. What advice would you give future principal investigators on partnering with clinicians?
   2. Is there anything else that you would like to add with regards to your experience of partnership with clinicians in the context of IKT?

Other open-ended questions

1. What comes to mind when we ask about your experience with Edith Strauss? Or what comes to mind when you think about your experience with Edith Strauss initiatives?
2. What is the difference between your experience and the ideal?
3. What surprised you the most about your experience?
4. Tell me about your experience collaborating with a clinician.
5. What was frustrating about the project? (or biggest challenge/problem encountered.

Also, biggest sources of satisfaction/most proud of/biggest success)

1. What have you learned?
2. What would you change?
