# Supplementary material for: A mixed-methods approach to understanding partnership experiences and outcomes of projects from an integrated knowledge translation funding model in rehabilitation
Source: BMC Health Serv Res. 2019 Apr 16;19:230. doi: 10.1186/s12913-019-4061-x (PMC6469130; doi:10.1186/s12913-019-4061-x)
Supplement: Supplementary file 3 — Interview protocol, this document presents the protocol used to facilitate the individual interviews with project leaders (DOCX 15 kb) [file 12913_2019_4061_MOESM3_ESM.docx]

Additional File 3: Interview Protocol

**CLINICIAN INTERVIEW**

**INTRODUCTION**

Our goal is to explore your experience of the partnership with a researcher and your perceptions of the impact of your participation in an [IKT initiative] research project on clinical practice. Ultimately, we wish to identify characteristics of successful partnerships that are most likely to impact clinical practice.

**GROUND RULES**

We would like to reiterate and reassure you that all comments and opinions provided today will remain confidential. Everything said here today will be transcribed, anonymously, and will be used solely for the purpose of coding qualitative data in regard to the project.

**QUESTIONS**

1. What comes to mind when we ask about your experience with Edith Strauss?
2. What were your expectations when you received funding to participate in the ES project?
3. Outcomes:
4. What were the impacts of your project?
5. How do you feel about it?
6. Why? *Why did the project have this impact?*
7. How has your clinical practice changed as a result of your project?
8. What did you learn from this experience?
9. How did this project influence your relationship with research in general?
10. Partnership:
11. Tell me about your experience working with a researcher
    - *Probe: What are some of the challenges that you encountered working with a researcher? (What was difficult? What was easy?)*
12. Who initiated the research project / and how did that set the dynamics for the partnership?
    - *Probe: How did you become involved in your ES project?*
    - *Probe: How was power sharing within the partnership?*
13. What would be an ideal partnership?
    - How does that compare to your actual experience?
    - *Probe: What factors do you attribute to this? What explains the difference?*
14. Motivations:
    1. What were your motivations to become involved?
       - *Probe: Why did you get involved? What incentives, if any, motivated you?*
    2. What is the best way to motivate clinicians?
       - *Probe: What is the best strategy to get clinicians involved?*
    3. How can we sustain clinician involvement in research?
       - *Probe: What is feasible in terms of sustaining participation?*
15. What research experiences have you had in the past?
16. **Final words:** What advice would you give to future clinicians participating in ES projects? Is there anything that we haven’t talked about that you’d like to share?
