# Supplementary material for: A mixed-methods approach to understanding partnership experiences and outcomes of projects from an integrated knowledge translation funding model in rehabilitation
Source: BMC Health Serv Res. 2019 Apr 16;19:230. doi: 10.1186/s12913-019-4061-x (PMC6469130; doi:10.1186/s12913-019-4061-x)
Supplement: Supplementary file 4 — Example of interpretive description coding and analysis, this document presents an example of the early coding process and the iterative reflections during analysis (DOCX 16 kb) [file 12913_2019_4061_MOESM4_ESM.docx]

Additional file 4: Example of interpretive description coding and analysis

| **Transcript Excerpt**  Interviewer: You’ve mentioned the kind of personal impact on you, how it helped you see the broader picture. Can you say more about the other impacts of the project on other areas?  Project leader (PL): The only thing is, we haven’t fully completed the project. The funding was only for a portion of it. So we never implemented the knowledge translation intervention we developed. So we never got to that step. So I cannot really about that step. I think that one of the impact is that going to conference, discussing the results that we obtained, being able to publish, partially, some of the results. We’re still trying to publish now. And I think we just went back to, we did it at the [name of institution], we went back to the [name of institution] and to the other site where we did it, to try to present, ok what were our findings. So I think it had a small impact on the managers and the actual clinicians. | **Early codes applied**  Project incomplete  Funding (for a portion of the whole project)  Implementation phase was not reached  Difficult to pronounce on actual impact on clinical practice  Impact/outcome of KT project:   - Publication (some results published, other in process) - Conference - Discussing results (general) - Presentation of findings to clinical site - Small impact on managers and clinicians (unsure though) |
| --- | --- |
| **Iterative reflections *(themes in italic)***  Funding only allowed for a portion of the project to be completed; the project did not reach implementation phase (*step in a larger effort*). PL seemed a bit disappointed about this and did not mention the sustainability plan (in this excerpt). For this reason, the PL was not able to pronounce on the impact on clinical practice (*difficulty in measuring impact*). The PL seemed confident that the project had an impact on managers and clinicians, even though she could not state exactly how so.  The PL was able to name outputs (*project deliverables*) of the project such as publication, disseminating results at conferences (*scientific contribution)* and at clinical sites. The PL states that these means of dissemination must have had an impact on the people receiving the information (*increased awareness of best practices),* however the PL was unsure of the extent of this impact *(difficulty measuring clinical changes).* | |
